# A phase 2 randomized trial to evaluate the impact of a supervised exercise program on cardiotoxicity at 3 months in patients with HER2 overexpressing breast cancer undergoing adjuvant treatment by trastuzumab: design of the CARDAPAC study

**DOI:** 10.1186/s12885-017-3420-4

**Published:** 2017-06-19

**Authors:** Quentin Jacquinot, Nathalie Meneveau, Marion Chatot, Franck Bonnetain, Bruno Degano, Malika Bouhaddi, Gilles Dumoulin, Dewi Vernerey, Xavier Pivot, Fabienne Mougin

**Affiliations:** 1 UPFR des Sports, Université de Franche-Comté, 31 chemin de l’Epitaphe, 25000 Besançon, France; 2 EA 3920: Marqueurs pronostiques et facteurs de regulation des pathologies cardiaques et vasculaires, CHU Jean-Minjoz, 25000 Besançon, France; 3 Service d’Oncologie Médicale, CHU Jean-Minjoz, 25000 Besançon, France; 4 Service de Cardiologie, CHU Jean-Minjoz, 25000 Besançon, France; 5 INSERM UMR 1098: Unité de méthodologie et de qualité de vie en cancérologie, CHU Jean-Minjoz, 25000 Besançon, France; 6 Physiologie-Explorations Fonctionnelles, CHU Jean-Minjoz, 25000 Besançon, France; 7 Laboratoire de Biochimie Endocrinienne et Métabolique, CHU Jean-Minjoz, 25000 Besançon, France

**Keywords:** Breast cancer, HER2 overexpression, Cardiotoxicity, Exercise, Study protocol, Supportive care

## Abstract

**Background:**

The overexpression of human epidermal growth factor receptor-2 (HER2) in breast cancer is a poor prognosis. Trastuzumab improves overall survival but is associated with cardiotoxicity, especially a decline in left ventricular ejection fraction (LVEF). In addition, chemotherapy and radiotherapy increase fatigue and pain, decrease physical capacity and health-related quality of life. To date, no study has evaluated the benefits of physical activity on the side effects of treatment in patients with HER2 positive breast cancer. The aim of this study is to evaluate the impact of 3 months’ exercise intervention on myocardial function and in particular on the rate of cardiotoxicity.

**Methods:**

This multicenter, randomized clinical trial will include 112 patients treated by adjuvant trastuzumab for HER2 positive breast cancer to investigate the effects of a 3 months’ supervised exercise program (intermittent exercise, combining moderate and high intensities; 55 minutes duration, 3 times per week), on the rate of cardiotoxicity [defined by either a decrease of the LVEF under 50% or an absolute drop of LVEF of 10%] between baseline and at 3 months and on strength, aerobic capacity, metabolic, inflammatory and hormonal parameters. Health-related quality of life, fatigue, pain and level of physical activity will also be assessed. Participants are randomly allocated to one of the two groups (“training group” vs “standard oncological care”). Performance-based and self-reported outcomes are assessed at baseline, at the end of supervised exercise program and at six months follow-up.

**Discussion:**

Although physical exercise is recommended to reduce the side effects of adjuvant treatments in breast cancer patients, no randomized study has been conducted to assess the benefits of a physical training program in patients with HER2 overexpressing breast cancer. Cardiac toxicity of trastuzumab may be minimized with an exercise program combining high and moderate intensities. This type of program may be safe, feasible and effective but also increase cardiorespiratory fitness and improve health-related quality of life. If these benefits are confirmed, this exercise intervention could be systematically proposed to patients during the course of treatment by trastuzumab in addition to standard oncological care.

**Trial registration:**

National Clinical Trials Number (NCT02433067); Registration 28 april 2015.

## Background

Breast cancer is the most frequently diagnosed cancer and the leading cause of cancer death among females [1]. In France, with 48,763 new cases reported in 2012, breast cancer represents 31.5% of all incident cancers in women, and almost 14% of all incident cancers in both sexes [2]. Breast cancer also causes more deaths in women, with 11,886 estimated deaths in France [2]. The overexpression of human epidermal growth receptor 2 (HER2) proteins concerns approximately one third of breast cancer patients [3, 4]. This overexpression has historically been associated with poorer disease-free and overall survival [3, 5]. However, targeted treatment using monoclonal antibodies against HER2 expression, such as trastuzumab, in addition to standard chemotherapy is associated with substantial improvements in disease-free survival and overall survival [6–8]. However, these agents are associated with cardiotoxicity but mechanisms are still unknown [3, 9]. Cardiotoxicity is the main side effect and is defined by either a decrease of the LVEF under 50% (this decrease was independent from the baseline value) or an absolute drop of LVEF of 10% [6, 10, 11]. Indeed, the rates of heart failure and asymptomatic decline of left ventricular ejection fraction (LVEF) have been reported to range from 0.4 to 4.1%, and 3 to 18%, respectively in this indication [7, 12]. In addition to cardiotoxicity, chemotherapy and radiotherapy also engender other side effects including weight loss or gain [13], fatigue [14], muscle wasting, reduction of physical fitness [15] as well as impaired exercise capacity with a VO_2_ peak reportedly 27% below age-matched healthy sedentary women [16]. This in turn can have negative impacts on activities of daily living and health-related quality of life [17].

Physical exercise programs are increasingly being recognized as an effective strategy to counteract the adverse effects of cancer therapy, such as a decline of cardiorespiratory fitness [18], muscle strength [19], immune function [20] and quality of life [21]. Nonetheless, to date, no consensus exists regarding the type and intensity of exercise that is most effective during treatment. Waart et al. [22] reported that low-intensity program may be easier for patients to follow during chemotherapy, whereas moderate-to high-intensity programs may be most effective in minimizing decline in cardiorespiratory fitness, muscle strength, and in limiting fatigue and symptom burden. To the best of our knowledge, only Haykowsky et al. [23] have investigated the effects of physical exercise on myocardial function in patients with HER2 positive breast cancer, and they showed that adjuvant trastuzumab therapy is associated with left ventricular (LV) dilation and a reduction in LVEF despite aerobic exercise training. According to the authors, the intensity of their program was inadequate as a stimulus to prevent LV remodelling. Indeed, the intensity of the exercise would be an important element to reshape the LV. High intensity activity (95% of maximum heart rate) would appear to be effective in remodelling of LV in patients with heart failure. [24] However, it’s difficult to know whether patients treated with chemotherapy and trastuzumab in adjuvant can perform and tolerate this high intensity exercise to remodel the LV.

Therefore, the purpose of this study is to evaluate, in patients with HER2 positive breast cancer and treated exclusively by trastuzumab, the impact on cardiac function (as assessed by LVEF) of an individualized, intermittent aerobic exercise training regimen (55 min, 3 times a week), comprising both moderate and high intensity exercise, for a period of 3 months. Secondary objectives are to evaluate the effect of this supervised exercise program on other parameters such as longitudinal strain, ventricular volumes and mass, body composition (lean and fat mass), cardiorespiratory fitness, quadriceps strength and metabolic, inflammatory and hormonal variables. Furthermore, quality of life, fatigue, pain and level of physical activity are also assessed.

We hypothesize that a supervised exercise program will maintain at a constant level or increase LVEF and improve myocardial parameters as longitudinal strain, volumes and mass of ventricles. Exercise increases quadriceps strength and cardio-respiratory fitness, with an improvement in metabolic, hormonal and inflammatory variables. We purport that these modifications will be accompanied by a decline in fatigue and pain, and by improved quality of life. Moreover, we hypothesize that patients who participate in the supervised exercise program will maintain these benefits at the three-month follow-up.

If this program confirms these beneficial effects, supervised exercise interventions could be systematically proposed to patients with HER2 positive breast cancer, in addition to standard oncological care, to reduce the side effects of trastuzumab and facilitate the return to social, family and professional life.

## Methods

**Fig. 1:**
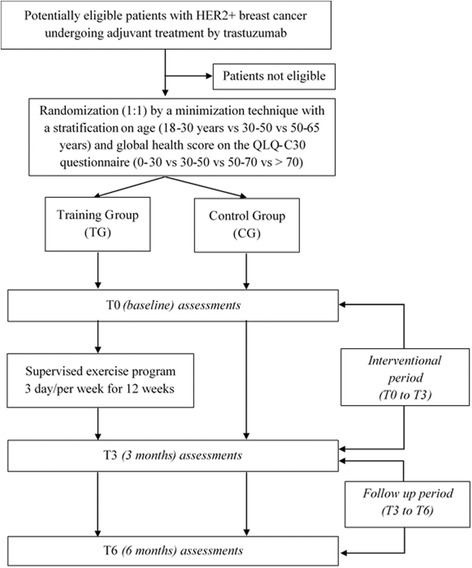
Flowchart of the study design

The design of the trial is displayed in Fig. 1. The study has been approved by the ethics committee and the National Health Products Safety Agency (P/2014/241). Patient recruitment and data collection started in April, 2015. Financial support for this work is provided by the Ligue Contre le Cancer (CCIR-GE).

### Recruitment and inclusion

**Fig. 2:**
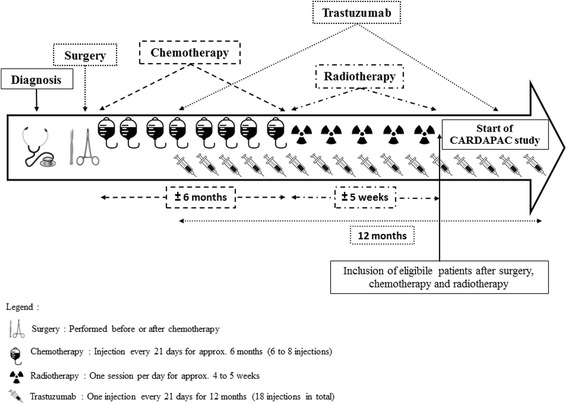
Representation of treatment schedule and study inclusion for patients with HER2 positive breast cancer

In total, 112 patients are being recruited for this study. All potentially eligible patients, followed-up in the department of medical oncology for HER2 positive breast cancer, are identified during multidisciplinary team meetings with oncologists, surgeons and radiotherapists in one of the eight public hospitals in the region of Franche-Comté (eastern France). The inclusion and exclusion criteria are detailed in Table 1. Eligible patients are informed about the study, and given an information leaflet. Before inclusion, patients with HER2–positive breast cancer must have undergone surgery, and chemotherapy plus radiotherapy if indicated. The choice of type of surgery, as well as the chemotherapy and radiotherapy regimens are left at the discretion of the physician. Patients are included when they receive adjuvant treatment with trastuzumab (administrated every 3 weeks for a total of 18 injections) (Fig. 2). At the inclusion visit, the signed informed consent is retrieved and a clinical examination is carried out. The medical history, use of analgesics (type, level, dose) and questionnaires on lifestyle habits (smoking, alcohol) are also recorded.

**Table 1:** Inclusion and exclusion criteria

Inclusion criteria	Exclusion criteria
- Patients aged 18 to 85 years - First HER2 positive breast cancer, confirmed histologically - WHO Performance status ≤1 - Completed chemo-radiotherapy - Normal renal function (creatinine clearance ≥60 ml.min-1) - Normal heart function with LVEF ≥50% (As assessed by echocardiography dating from less than 3 months previously) - Normal liver function (normal ASAT and ALAT) - Certificate of non-contraindication to the practice of physical activity - Active contraception or menopaused	- HER2 negative Breast cancer - Patients with metastases - Heart failure (LVEF ≤50%) - Resting oxygen saturation (SaO_2_) ≤ 92% - Autoimmune disease (systemic lupus erythematosus, rheumatoid arthritis) - Symptomatic osteoarthritis, cardiovascular disease (angina or uncontrolled hypertension) or lung disease (chronic obstructive pulmonary disease) - Patients suffering from malnutrition (body mass index <18 kg.m^−2^) or weight loss of >10% during the previous 3 months - Patients with psychiatric or cognitive disorders deemed unsuitable for physical activity - Pregnant or breastfeeding patients

### Randomization

After signing the consent, patients are randomized to the Training Group (TG) or the Control Group (CG). Randomization is conducted in open manner, in a 1:1 ratio and performed according to the minimization technique with stratification (eRandomisation software Tenalea®) by age (18–30 years vs 30–50 vs 50–65 years) and global health score on the QLQ-C30 questionnaire (0–30 vs 30–50 vs 50–70 vs > 70).

### Study outcomes

All evaluations are carried out at inclusion (T0), and at three (T3) and 6 months (T6) of the follow up period. Between T0 and T3, both groups followed standard oncological care either without (control group: CG) or with (training group: TG) a supervised exercise program (3 times/week). During the follow-up period (T3-T6), both groups followed standard oncological care but no supervised physical activity. An assessment of a level of physical activity for 7 days by an actimeter is achieved between T0-T3 and T3-T6 without guidelines is imposed.

### Primary endpoint

Cardiac toxicity is defined by either a decrease of the LVEF under 50% (this decrease was independent from the baseline value) or an absolute drop of LVEF of 10% from baseline. LVEF is assessed by a transthoracic doppler echocardiography and is performed at T0, T3 and T6 according to the American Society of Echocardiography recommendations [25] with a Philips echocardiography machine (Philips iE 33 or EPIQ7, Philips Healthcare, Andover, MA, USA) and a 2.5 MHz probe. Measurements are made on 3 representative beats and the mean of the results is recorded. Standard echocardiographic analysis included two-dimensional, M-mode, and Doppler flow measurements. All echocardiograms are carried out by the same observer, who is blinded to the clinical data. LVEF is measured in the apical 4- and 2-chamber views using Simpson’s biplane rule and with TM measurement in 2D mode in the left parasternal view according to the American Society of Echocardiography guidelines [26].

### Secondary endpoints

#### Other myocardial and valve function

All other measurements are assessed by transthoracic Doppler echocardiography as described above according to the American Society of Echocardiography guidelines [25]. 2D images are acquired in the apical and parasternal axes and subcostal view, with the latter also used for M-mode imaging. Valve regurgitation is assessed and graded in accordance with consensus criteria based on Doppler and M-mode imaging [27, 28]. LV mass is calculated as the product of myocardial density and myocardial volume. Peak systolic longitudinal strain is calculated by averaging the values of peak systolic strain in the basal, midventricular and apical segments in the 4 and 2-chamber views [29]. Left and right ventricular fractional shortenings are calculated as the percentage drop in right ventricular outflow tract diameter in systole with respect to diastole [30].

#### Anthropometric and body composition

The height is determined to the nearest 0.01 m using a calibrated length board. Body mass is measured to the nearest 0.1 kg using a calibrated. Body mass and height are measured bare-foot while wearing underwear. Body mass index (BMI) is calculated as body mass divided by height squared (kg.m^−2^). Waist circumference (WC) is measured to the nearest 0.5 cm in a standing position with a standard non-elastic tape that was applied horizontally midway between the last rib and the superior iliac crest. Body composition is determined by the skinfold method (Harpenden® skinfold caliper, Baty International, Burgess Hill, England). The measurement is performed by the same operator 3 times on the same fold (the fold biceps, supra iliac fold, fold subscapularis, the triceps fold) and the average of 3 values is taken into account. The body composition also is evaluated on fasting subjects, lying flat for 15 min, not having drunk and having performed no physical effort for at least 12 h by multifrequency (5, 50, 100, 200 kHz) bioelectric impedance (Z-Metrix®, BioparHom, Bourget du lac, France), a simple, rapid, and non-invasive assessment.

#### Resting respiratory function

Resting respiratory function is explored by the analysis of breathed air. All pulmonary function tests are carried out by the same technician following the guidelines of the American Thoracic Society [31] using a spirometer (CPFS/D, Medical Graphics, Strasbourg, France) which is a device that measures the flow rate of instantaneous gas mouth open circuit. It therefore measures the flow rates and the patient’s lung volumes. For this study, a flow-volume loop is performed during which the following parameters are evaluated: forced vital capacity (FVC), forced expiratory volume in one second (FEV1), FEV1/FVC ratio, and maximal expiratory flow (MEF) at 25%(MEF_25_), 50% (MEF_50_), 75%, (MEF_75_) and 25–75% (MEF_25–75_) of FVC. These data are necessary before a maximal graded test.

#### Cardiorespiratory fitness

Patients performed a maximal graded exercise test, under a cardiologist’s supervision, using a cycle ergometer (Ergoselect 200; Ergoline; Bitz, Germany) at T0, T3 and T6. After an initial warm-up period of 3 min (30 watts), exercise begins at a power output of 10 W every minute until exhaustion. The cadence is maintained between 50 rpm and 70 rpm. Multichannel electrocardiograms (ECGs) (CASE P2, GE Healthcare, Buckinghamshire, UK) are monitored online before, during exercise and recovery to follow heart rate. During exercise, the subjects were connected to a gas analyzer system (MGC-CPX System; MGC Diagnostics Corporation, Saint Paul, MN, USA), which was calibrated using gases of known concentration. Expiratory gases were sampled and analyzed for each 20 s period. The variables determined are: rate of oxygen consumption (*V˙O*_2_) and carbon dioxide production (*V˙*CO_2_), respiratory exchange ratio (*V˙*CO_2_/*V˙O*_2_), and ventilation per minute (*V˙*E). The ventilatory threshold 1 (VT_1_) and 2 (VT_2_) are assessed from *V˙*E, the relation between *V˙*CO_2_/*V˙O*_2_, *V˙*E⁄*V˙O*_2_, *V˙*E⁄*V˙CO*_2_ and power output by three experts in a blind fashion using the V-slope method. The mean of the two closest values is taken as the ventilator threshold and the corresponding power output (*W˙VT*) was registered [32] and mechanical power (Watts) corresponding to the ventilatory threshold 1 and 2 (VT_1_ and VT_2_) are used for the rehabilitation.

Blood gas (SaO_2_, PaO_2_, PaCO_2_, pH and Excess Bases) and lactate levels are collected by microwave method on a sample taken by incision of the earlobe at rest, at maximal effort and after 5 min recovery [33]. To confirm that exhaustion is reached, two of the three following criteria must be met: a drop in cadence below 50 rpm, a respiratory exchange ratio value exceeding 1.0, attainment of 100% of age-predicted maximal HR (220-age).

#### Maximum voluntary force of the leg extensor

Maximum voluntary strength of quadriceps is performed sitting, with the knee flexed to 90° with a strain gauge (SENSY’s load cell 2712). The force was measured on the right leg and then the left leg, only the value of the dominant leg was reported. To obtain a valid measurement, it is necessary that the patient’s strength reaches a plateau and is maintained at least 5 s. Three repeats are performed and the best of the three is retained. The signal from the strain gauge is sent to a Power lab 26 T® series with dual bioamplifier (AD Instruments United Kingdom, model No. ML4856) and the data are analyzed with LabChart 8 Pro Software®.

#### Biological and hormonal parameters

Blood samples are collected in the morning (8:00 to 10:00) after an overnight fast, by venous puncture. Plasma is separated by centrifugation (15 min at 3500 rpm) and aliquots are stored at −80 °C until biochemical analysis. Plasma glucose, cholesterol, HDL-cholesterol, LDL-cholesterol, triglycerides, C-reactive protein (CRP), interleukin-6 (IL-6), tumor necrosis factor-alpha (TNF-α), insulin, insulin-like growth factor (IGFs), leptin and adiponectin are measured. Homeostatic model assessment (HOMA) is calculated as [fasting insulin (mU/l) x fasting glucose(mmol/l)/22.5] and used to estimate insulin resistance [34].

#### Questionnaires

All questionnaires are completed prior to the completion of each maximal graded test (T0, T3 and T6).

##### Health-related quality of life and body image

The cancer-specific Health-related Quality of Life Questionnaire EORTC QLQ-C30 is a self-administered, validated questionnaire to assess HRQoL in cancer patients [35]. It contains 30 items covering five functional scales (physical, role, cognitive, emotional, and social), three symptom scales (fatigue, pain, and nausea and vomiting), and a global health and quality-of-life scale. The remaining single items assess additional symptoms commonly reported by cancer patients (dyspnea, appetite loss, sleep disturbance, constipation, and diarrhea), as well as the perceived financial impact of the disease and treatment.

In addition, the 23-item breast cancer specific module EORTC QLQ-BR23 is completed, assessing 8 dimensions specific to breast cancer patients: four functional scales (body image, sexual functioning and enjoyment, future perspective) and four symptomatic scales (arm symptoms, breast symptoms, systemic therapy side effect and hair loss). For the present study, only the body image items are compiled.

##### Fatigue

The French version “Multidimensional Fatigue Inventory 20” (MFI 20) validated by Gentile et al., (2003) is a 20-item self-report instrument, designed to measure fatigue [36]. It covers the following dimensions: general fatigue, physical fatigue, mental fatigue, reduced motivation and reduced activity. This instrument was tested for its psychometric properties in cancer patients receiving radiotherapy. Patients assess themselves on a scale of 5 levels, from 1 to 5, according to the fatigue experienced the day before the questionnaire was completed.

##### Pain

The Brief Pain Inventory short form (BPI-SF) is a validated, widely used, self-administered questionnaire developed to assess the pain severity and pain interference during the previous 24 h [37]. The BPI-SF includes diagrams of the front and back of the body, four items to capture the variability of pain over time: pain at its “worst,” “least,” “average,” and “now” (current pain), the seven items relating to the interference of pain with various daily activities, including general activity, walking, work, mood, enjoyment of life, relations with others and sleep rated on 0–10 scale, as well as a question about percentage of pain relief by analgesics.

#### Level of physical activity

The International Physical Activity Questionnaire (IPAQ) questionnaire and an actimeter (Actigraph®, Fort Walton Beach Florida, USA) are used to evaluate the level of physical activity.

The IPAQ validated in French assesses overall physical activity the last 7 days (leisure time physical activity, sport, physical activity at work, activities of daily living, transportation) in adult populations aged 15 to 69 years [38]. The items are structured to provide separate scores on walking, moderate-intensity and vigorous-intensity activities as well as a combined total score to describe overall level of activity. Responses are converted to Metabolic Equivalent Task minutes per week (MET.min.week^−1^) according to the IPAQ scoring protocol [39].

The Actigraph® allows measurement of the overall activity of an individual in the activities of daily living. It is a small accelerometer which measures accelerations from 0.05 to 2.00 G [40]. These accelerations are scored in counts per minute that provide information about the duration and intensity of activity. Patients wear the accelerometer on the right hip for 7 consecutive days during the period T0-T3 and between T3-T6. The Actigraph® does not give any form of feedback to the participants.

### Supervised exercise program

**Fig. 3:**
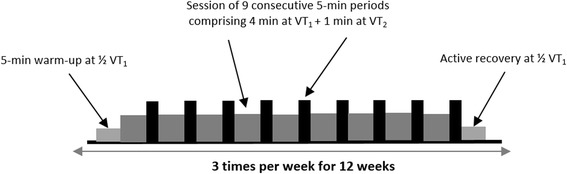
Supervised exercise program

The supervised exercise program consists of three 45-min exercise sessions a week for 12 weeks. Each training session includes 9 successive work bouts of 5 min each. During each work bout, a 4-min period of moderate work (base) is followed by a 1-min period of intense work (peak). Initially, the base is set at the first ventilatory threshold (VT_1_) obtained on the initial maximal exercise test, and the peak is set at the second ventilatory threshold (VT_2_). The program starts with a 5-min warm-up at intensity equal to ½ VT_1._ Then, the intensity of each training session is designed to lead up to almost 80% of maximum heart rate at the end of the peak. The peak and base loads are alternately readjusted by 10 W when the heart rate recorded at the end of the session is 10 to 12 beats/min below the target heart rate. Hence, each training session corresponds to the maximal endurance intensity that the subject is able to maintain for 45 min. Active recovery is carried out at ½ VT_1_ for 5 min (Fig. 3).

### Sample size

The primary objective of this phase II, randomized, prospective, multicenter; non-comparative trial is to evaluate the rate of cardiotoxicity at 3 months (a decrease of the LVEF under 50% or an absolute drop of LVEF of 10% from baseline) of patients with HER2 overexpressing breast cancer undergoing adjuvant treatment by trastuzumab. According to Fleming one-stage design with a one-sided 5% type I error and power of 90%, 53 patients in the training arm will need to be randomized in order to test the following hypotheses:

- H0 (null): a cardiotoxicity rate at 3 months of 75% (uninteresting to pursue any further investigation)
- H1 (alternative): a cardiotoxicity rate at 3 months of 90% (warrants further investigation in a phase III trial).

The hypotheses regarding an anticipated cardiotoxicity rate at 3 months of 90% and an uninteresting rate of 75% is based on the observed cardiotoxicity rate in randomized clinical trials who has been to be approximately 13 to 27% according the molecules used for chemotherapy [9]. The usual care arm will serve as calibration that the populations in the two arms are similar and to validate the H0 hypothesis: no statistical comparison is planned between the two arms.

In the training group, after recruitment of the 53 patients with a 3-month follow-up from randomization:

- if 44 or less than 44 patients are free of cardiotoxicity at 3 months (83.0%), the supervised exercise provided in the training arm could be declared uninteresting.
- if 45 or more than 45 patients are free of cardiotoxicity at 3 months (84.9%), the supervised exercise could be declared interesting for further phase III evaluation.

The probability to conclude for inefficacy at the end whereas *p* = 90.0% is β = 7.8%. The probability to conclude for efficacy at the end whereas *p* = 75.0% is α = 6.1%.

Overall, 53 patients will be included in both arms: 106 patients need to be randomized. With an expected 5% rate of patients not evaluable at 3 months or drop out patients, it will be necessary to include a total of 112 (106*1.05) patients (Training arm: *N* = 56; Usual care arm: *N* = 56).

### Statistical analyses

A final statistical plan will be written before data frozen. A specific statistical plan dedicated to HRQoL analyses will be also written before data frozen. The statistical analyzes will be carried out with software SAS® v9.2 (SAS Institute Inc., Cary, NC, USA). Clinical and demographic data will be described using rules form. The statistical parameters mean, median, SD, interquartile range and range will be presented for continuous baseline variables. For categorical baseline variables, corresponding frequencies (n, %) will be calculated. All baseline variables will be summarized by treatment arm. Means and medians will be compared using Student’s t-test and Wilcoxon test, respectively. Proportions will be compared with Chi2 test (or Fisher’s exact test, if appropriate).

The primary analysis will be performed in modified intention-to-treat (mITT) population, i.e. including all evaluable randomized patients regardless of their eligibility and supervised exercise received. The results will be reported according to the randomized treatment.

Confirmative analyses will be conducted firstly in the ITT population (not assessable patients and patients with drop out between randomization and 3 months will be considered as progressive) and secondly, in the Per Protocol population defined as patients who have received at least a part of the supervised exercise and presenting no major deviations from the protocol.

Patient compliance to the supervised exercise program will also be evaluated by the ratio of the total number of imposed sessions (*n* = 36) to the number of really performed sessions.

Correlation analyses will be performed to determine whether the level of cardiorespiratory fitness is significantly associated with the changes in cardiac function over time (longitudinal strain, volume), muscle strength, fatigue, pain, quality of life and metabolic, hormonal and inflammatory responses. Similarly, we will investigate whether program effectiveness varies significantly as a function of patients’ background characteristics (age, weight, type of chemotherapy), and particularly those variables assessing level of activity, and patient compliance to the supervised exercise program.

The time to HRQOL score deterioration will be estimated as a modality of longitudinal HROQL analysis. It will be defined as the time from inclusion in the study to the occurrence of the first clinically significant deterioration of 5 point at least of the HRQOL score as compared to the baseline score [41]. Dimensions targeted will be the physical functioning, emotional functioning and fatigue scales of the QLQ-C30. All other dimensions of HRQOL will be also analyzed. Patients with no follow-up measure will be censored just after baseline (Day 1). Patients with no deterioration before their drop-out are censored at the time of the last HRQoL assessment. TTD curves were estimated using the Kaplan-Meier method and described using median and its 95% CI. Some univariate and multivariate analysis will be performed as exploratory purpose only in order to investigate factors potentially influencing the TTD. Other definitions of TTD will be explored as sensitivity purpose varying the MCID and including death as an event.

## Discussion

Several studies have highlighted the benefits of physical activity during adjuvant treatments in breast cancer in reducing side effects, and have reported an improvement in cardiorespiratory fitness [18, 42, 43], muscle strength [18, 19], immune-function [20], body composition [44, 45] as well as quality of life [21] and fatigue [21, 46–48].

To the best of our knowledge, CARDAPAC is the first randomized study to assess the effect of a supervised exercise program on the incidence of cardiotoxicity induced by trastuzumab in patients with HER2 positive breast cancer. Only Haykowsky et al. [23] have demonstrated left ventricular dilation and a reduction in LVEF in HER2 positive breast cancer despite aerobic exercise training. Furthermore, theses authors did not observe an improvement in exercise capacity (power output, VO_2_, heart rate, perceived effort) after the aerobic training intervention performed during the first 4 months of trastuzumab therapy, 3 days per week, for between 30 min to 60 min. They suggest that the intensities of the aerobic program (heart rate equal to 60% to 90% of peak oxygen consumption) and the number of sessions were not only insufficient to prevent left ventricular remodelling, but also to achieve beneficial training adaptations. However, this type of aerobic training program could prevent the expected decline in peak oxygen consumption that occurs during the first 4 months of trastuzumab treatment, as previously reported by Peels et al. [49].

Unlike the study of Haykowsky et al., in which the exercise programme took place during the first 4 months trastuzmab therapy, our study includes patients at the end of their adjuvant treatments (chemotherapy and radiotherapy) and consequently limits potential bias from the side effects of these treatments. Furthermore, it also includes two randomized arms with a control group, and a training group who perform a supervised, individualized, intermittent exercise regime combining both moderate and high intensities, over a period of 3 months. The CARDAPAC study is the first randomized controlled trial in this indication, and has several strengths, including the multicentre nature of the trial, the large sample size, and the supervised, individualized exercise program.

Given the lack of recommendations for exercise during adjuvant breast cancer treatments (particularly regarding type, duration, or frequency), we propose a specific endurance training in our training group called the Square-Wave Endurance Exercise Test (SWEET) validated by Gimenez et al. [50]. This bi-level training aims to replicate interval training sessions (moderate and high intensity) adjusted according to the physical characteristics of the subjects. It has previously been proposed with healthy subjects [50], but also with patients suffering from cardiovascular [51, 52] or other diseases [53, 54]. SWEET is quite efficient in increasing maximal oxygen consumption, endurance capacity, and/or force and endurance of the leg muscles.

This study was designed to reduce the side effects of previous adjuvant therapies and the cardiotoxicity of trastuzumab in keeping at a constant level or in increasing of the LVEF. In addition, it meets the goals of supportive care, which are to improve quality of life during treatment and facilitate the post-treatment phase with maintenance of the program benefits in the long term.

Finally, in case beneficial effects are observed at the end of the exercise intervention, it is of interest to investigate whether these benefits are sustained over a longer period of time. Therefore, we intend to follow-up all patients for 3 months after the exercise intervention to assess physical fitness, fatigue, quality of life and level of physical activity.

It should be noted that during the study the level of physical activity and body composition are assessed which may lead patients to modify their eating habits and lifestyle (level of physical activity). Nevertheless, we do not anticipate that this will take place in a structured or systematic way, and thus the planned comparisons (between the training groups and the control group) will still be valid.

To summarize, the use of targeted therapies in the treatment of cancer in constantly increasing. Substantial improvements in disease-free and overall survival have been reported with these therapies, particularly trastuzumab. Although there are fewer side effects than with the surgery, chemotherapy and radiotherapy must not underestimate the side effects of these targeted therapies.

If this study shows that physical activity, combining high and moderate intensities, can reduce or minimize the cardiotoxicity of trastuzumab, increase cardiorespiratory fitness and health-related quality of life. This exercise intervention could be systematically proposed to patients with HER2 positive breast cancer in addition to standard oncological care.

## Abbreviations

2D mode: Two-dimensionel doppler echocardiography
ALAT: Alanine aminotransferase
ASAT: Aspartate transaminase
BMI: Body mass index
BPI-SF: Brief pain inventory-short form
BR23: The 23-item breast cancer specific module
CG: Control group
CRP: C-reactive protein
ECGs: Electrocardiogram
EORTC: European organization for research and treatment of cancer
FEV: Forced expiratory volume
FEV1: Force expiratory volume in 1 s
FVC: Vital capacity
HDL-cholesterol: Hight density lipoprotein
HER2: Human epidermal growth factor receptor 2
HOMA: Homeostatic model assessment
HR: Heart rate
HRQOL: Health-related quality of life
IGFs: Insulin-like growth factor
IL-6: Interleukin-6
IPAQ: International physical activity questionnaire
ITT: Intention-to-treat
LDL-cholesterol: Low density lipoprotein
LV: Left ventricular
LVEF: Left ventricular ejection fraction
MCID: Minimal clinically importante difference
MET.min.week^−1^: Metabolic equivalent task minutes per week
MFI 20: Multidimensional fatigue inventory 20
mITT: modified intention-to-treat
PaCO_2_: Partial pressure of carbon dioxide in the arterial blood
PaO_2_: Partial pressure of oxygen in arterial blood
pH: Hydrogen potential
QLQ-C30: Quality of life questionnaire core 30
SaO_2_: Oxygen arterial saturation
SD: Standard deviation
SWEET: Square-Wave Endurance Exercise Test
TG: Training group
TM: Time-motion mode
TNF-α: Tumor necrosis factor-alpha
TTD: Time to deteriation
VCO_2_: Carbon dioxide flow
VE: Ventilation rate
VE/VCO_2_: Respiratory equivalents in carbon dioxide
VE/VO_2_: Respiratory equivalent in oxygen
VO_2_ peak: Peak oxygen uptake
VO_2_: Oxygen consumption
VT_1_ and VT_2_: Ventilatory thresholds 1 and 2
W: Watts
W˙VT: Power output
WC: Waist circumference
WHO: World health organization
